# Risk-Stratified Interventional Management and Surgical Decision-Making in Primary Glaucoma

**DOI:** 10.3390/biomedicines14091983

**Published:** 2026-09-02

**Authors:** Chun Hsiung, Chiao-Hsin Lan, Wei-Ting Yen, Da-Wen Lu

**Affiliations:** 1 Department of Ophthalmology, Tri-Service General Hospital, Taipei 114202, Taiwan; drchunhsiung@gmail.com; 2 Department of Ophthalmology, College of Medicine, National Defense Medical University, Taipei 114202, Taiwan; 3 Department of Medical Education, National Taiwan University Hospital, Taipei 100225, Taiwan; sallylan410486@gmail.com

**Keywords:** primary open-angle glaucoma, primary angle-closure disease, risk stratification, intervention, selective laser trabeculoplasty, minimally invasive glaucoma surgery, trabeculectomy, tube shunt, normal-tension glaucoma, artificial intelligence

## Abstract

Primary glaucoma is a leading global cause of irreversible visual impairment, necessitating management strategies to preserve visual function over a lifetime. This narrative review synthesizes contemporary evidence on risk stratification, target intraocular pressure (IOP), and interventional decision-making for primary open-angle glaucoma (POAG) and the primary angle-closure disease (PACD) spectrum. A risk-stratified approach integrates baseline risk factors, gonioscopic anatomy, structural optical coherence tomography (OCT), standard automated perimetry, rate of progression, life expectancy, ocular surface status, medication adherence, patient preference, and resource setting. Early laser or surgical intervention can reduce medication burden and improve IOP control in selected patients. However, conservative medical management and observation remain appropriate when progression risk is low or procedural risk is disproportionate. Landmark trials support first-line selective laser trabeculoplasty in suitable POAG. Additionally, evidence supports primary trabeculectomy as an option for advanced disease and early lens extraction for selected older patients with primary angle closure or primary angle-closure glaucoma. Artificial intelligence, polygenic risk scores, and sustained-release platforms serve as promising adjuncts. Nevertheless, these technologies require external validation, equitable implementation, and clearly defined clinical action thresholds before routine adoption. Overall, glaucoma care should transition from a uniform sequence prioritizing topical medications toward an individualized, evidence-based escalation strategy. This approach optimally balances visual prognosis, procedural risk, and patient-centered outcomes.

## 1. Introduction

Glaucoma encompasses a group of progressive optic neuropathies characterized by retinal ganglion cell (RGC) apoptosis and irreversible visual field defects. As a leading cause of irreversible blindness worldwide, it represents a significant public health burden [1]. Epidemiological data project that its global prevalence will reach nearly 112 million by 2040 [2]. While primary open-angle glaucoma (POAG) is the most prevalent subtype, primary angle-closure glaucoma (PACG) also drives significant global visual morbidity, with projections estimating over 26 million affected patients by 2050 [3]. Despite expanded diagnostic modalities and therapeutic options, a subset of patients experiences clinically meaningful visual field progression even under active ophthalmologic care. Lowering intraocular pressure (IOP) currently represents the sole modifiable risk factor proven to slow the progression of glaucomatous optic neuropathy.

Traditionally, IOP lowering followed a reactive sequence beginning with topical hypotensive medications, with incisional filtering surgery reserved for eyes that failed maximum tolerated medical therapy or exhibited severe progression [4,5,6]. This approach remains appropriate for many patients, particularly when risk is low and adherence is reliable. However, chronic drop therapy is frequently compromised by nonadherence, inefficient drug delivery, multi-drop complexity, preservative exposure, ocular surface disease, cost, and reduced quality of life (QoL) [7,8,9,10,11]. Consequently, glaucoma care is increasingly incorporating an interventional framework in which selective laser trabeculoplasty (SLT), sustained-release delivery, lens-based procedures, minimally invasive glaucoma surgery (MIGS), trabeculectomy, or tube shunt surgery may be considered earlier when the expected benefit outweighs procedural risk [7,10,11,12,13].

With the rapid expansion of options, from tissue-sparing ab interno MIGS to traditional subconjunctival filtration, decision-making has grown increasingly complex [12,14]. In this review, the term “interventional” denotes laser, sustained-release drug delivery, lens-based, MIGS, and incisional approaches; the term “surgical” is reserved for lens and incisional procedures. A rigorous framework requires structural and functional progression metrics, anatomic phenotype, treatment burden, patient preferences, and local resource constraints [14,15,16]. This narrative review synthesizes current literature and expert consensus on primary glaucoma risk stratification, unified disease staging, and interventional decision-making with the goal of supporting individualized target IOP and timely escalation without overtreatment.

## 2. Literature Search Strategy

A narrative literature search was performed in PubMed and Embase. Search terms included combinations of ‘glaucoma’, ‘primary open-angle glaucoma’, ‘primary angle closure’, ‘risk stratification’, ‘target intraocular pressure’, ‘selective laser trabeculoplasty’, ‘minimally invasive glaucoma surgery’, ‘trabeculectomy’, ‘tube shunt’, ‘lens extraction’, ‘artificial intelligence’, ‘polygenic risk score’, and ‘consensus’. Articles published from 2000 to March 2026 were eligible, while randomized trials, systematic reviews, large longitudinal cohorts, formal guidelines, and consensus statements were prioritized. Because this is a narrative review rather than a systematic review, no pooled effect estimates were calculated. Evidence was interpreted by considering study design, population, follow-up duration, external validity, clinical applicability, and consistency with guideline recommendations. Because this was a narrative review, a PRISMA-style study flow, duplicate independent screening, and formal risk-of-bias assessment were not undertaken.

## 3. Comprehensive Risk Stratification in Primary Glaucoma

### 3.1. Baseline Clinical and Demographic Risk Factors

Accurate risk stratification in POAG and PACG necessitates evaluating both demographic variables and clinical parameters [15]. Large population studies identify advancing age, African or Hispanic descent, and a positive family history as principal non-modifiable risk factors for POAG [16,17]. Elevated IOP represents the primary modifiable risk factor driving disease progression [18,19]. Large-scale investigations, such as the Ocular Hypertension Treatment Study (OHTS), demonstrated that initial IOP and reduced central corneal thickness (CCT) serve as critical independent predictors of the development and worsening of glaucoma [20]. Other recognized risk factors for POAG are summarized in Table 1.

The risk factors associated with the onset and progression of primary angle-closure disease (PACD) spectrum encompass a complex interplay of demographic, anatomical, genetic, and dynamic physiological variables, as summarized in Table 2. Gonioscopy is the central diagnostic examination for confirming angle status and identifying peripheral anterior synechiae (PAS), while anterior-segment optical coherence tomography (AS-OCT) or ultrasound biomicroscopy can supplement but not replace clinical angle assessment [35]. Landmark longitudinal data from the Zhongshan Angle-Closure Prevention (ZAP) trial and the Singapore Asymptomatic Narrow Angle Laser Iridotomy Study (ANA-LIS) demonstrate that progression from asymptomatic primary angle-closure suspect (PACS) to manifest primary angle-closure (PAC) or PACG is remarkably low [38,39]. When clinical progression does occur, it is most frequently driven by the insidious development of peripheral anterior synechiae rather than acute IOP elevations, with older age and higher post-dilation IOP serving as the primary predictors of progression [38].

### 3.2. Structural and Functional Progression Metrics

Evaluating the rate of disease progression relies on longitudinal assessments of structural damage and functional loss. Recent studies emphasize that applying unified staging classifications to both POAG and chronic PACG is crucial, and functional defects for both can be reliably staged using identical methods [50]. Standard automated perimetry remains the foundational tool for functional monitoring and detecting glaucomatous visual field defects [51,52]. Visual field indices, such as mean deviation (MD) and the visual field index (VFI), quantify the rate of functional deterioration over time [35,51]. Specifically, the modified Glaucoma Staging System (mGSS), based on the VFI, is highly recommended for use in busy clinical settings for both POAG and PACG due to its simplicity and accuracy [50]. However, relying solely on functional data is insufficient because structural neuronal death often precedes detectable perimetric defects [1,16].

OCT provides objective quantification of the retinal nerve fiber layer (RNFL) and ganglion cell complex (GCC) thickness [7,35]. Combining structural and functional assessments is imperative for detecting early disease worsening [16,53,54]. Conversely, because structural OCT imaging eventually reaches a measurement floor effect in advanced disease where further tissue loss is no longer detectable, perimetry becomes the primary metric for continued monitoring in late stages [35,52,55]. To operationalize this principle, the latest consensus emphasizes integrating OCT data with functional metrics to prevent the underestimation of disease severity [56]. Systems like the Global Glaucoma Staging System (GGSS) simultaneously assess overall damage by plotting visual field loss against RNFL damage on continuous numerical scales [57]. Furthermore, unsupervised machine learning algorithms are now deployed to establish objective, age-adjusted RNFL thickness thresholds, grading structural risk strictly based on objective data rather than subjective interpretation [58]. Media opacity, particularly cataracts, can depress perimetric indices and mimic functional progression; cataract status should therefore be considered when reconciling structural–functional discordance and selecting lens-based or glaucoma surgery [35,52].

### 3.3. Unified Clinical Staging and Risk Stratification Frameworks

While individual metrics track progression, clinical management requires synthesizing these data points into standardized severity stages [58]. The evolution from a normal eye to glaucomatous blindness represents a continuum where structural changes generally precede functional visual field deficits [57,58]. Consequently, modern guidelines, such as the American Academy of Ophthalmology Preferred Practice Patterns, categorize disease severity by correlating structural imaging with standard automated perimetry (SAP) [16,56]. Notably, recent studies validate that these functional staging classifications can be reliably applied to both POAG and PACG [50].

- Preperimetric and mild (early) glaucoma: Patients exhibit definite structural abnormalities consistent with glaucoma (e.g., optic disc notching or thinning of the circumpapillary RNFL and macular GCC) but maintain a strictly normal visual field on SAP [16,56,57]. This is known as the preperimetric stage, where OCT identifies early neuronal loss before functional decline occurs. Utilizing the Hodapp–Parrish–Anderson (HPA) criteria, early functional loss is defined by an MD >−6 dB, with no sensitivity loss <15 dB within the central 5 degrees [59]. Objectively, artificial intelligence (AI)-enabled OCT models define early structural risk by a global RNFL thickness between 86.5 and 95.9 µm [58].
- Moderate glaucoma: This stage is characterized by definite structural damage alongside early visual field abnormalities localized to one hemifield that do not extend within 5 degrees of central fixation [56]. Under HPA criteria, the MD ranges between −6 dB and −12 dB [59]. AI thresholds categorize moderate structural severity as a global RNFL thickness between 71.5 and 86.4 µm [58].
- Severe (Advanced) glaucoma: This stage is defined by profound structural damage accompanied by visual field abnormalities in both hemifields or significant loss extending within 5 degrees of central fixation [56]. The HPA criteria designate this stage by an MD <−12 dB or at least one point in the central 5 degrees with a sensitivity of 0 dB [59]. Structurally, it correlates with a global RNFL thickness <71.5 µm and an advanced Disc Damage Likelihood Scale (DDLS) score ≥8 [58,60]. In end-stage disease, severe central scotomas often compromise fixation, rendering SAP unreliable and necessitating visual acuity impairment (e.g., ≤20/200) as the primary descriptor of functional damage [60].

While PACG shares the aforementioned functional staging with POAG, the PACD spectrum requires prerequisite anatomical staging based on the extent of iridotrabecular contact (ITC) [35]. The natural history is divided into distinct phases:

- PACS: ≥180 degrees of ITC with normal IOP and no neuropathy.
- PAC: ≥180 degrees of ITC with elevated IOP but no neuropathy.
- Definitive PACG: Manifest glaucomatous optic neuropathy and visual field loss.

To manage clinic flow and prioritize interventions, institutions combine these severity stages with broad clinical stratification matrices. For instance, the GLAUC-STRAT tool groups advanced POAG and advanced PACG into the highest risk category [15,50,61]. Furthermore, strict clinical red flags identify patients with a high-risk progression trajectory. These include rapid visual field deterioration (losing >2 decibels per year), acute IOP elevation >40 mmHg, having only one functional eye, or unexplained visual acuity loss [50,61]. Beyond these clinical flags, multivariable models highlight that a baseline IOP ≥ 25 mmHg, an advanced vertical cup-to-disc ratio (CDR) (≥0.9), and severe socioeconomic limitations (e.g., living on ≤US$2/day) are major independent predictors of the development of severe, end-stage functional glaucoma [60].

### 3.4. Advanced Risk Prediction Models

Modern glaucoma management increasingly incorporates risk calculators, triage tools, polygenic risk scores (PRSs), and AI models to predict individual trajectories [62,63,64]. The European Glaucoma Prevention Study and Ocular Hypertension Treatment Study (EGPS-OHTS) calculator is transparent and useful for ocular hypertension but less informative for established glaucoma, PACD, normal-tension glaucoma (NTG), or populations unlike its derivation cohorts [19,33]. Structural–functional systems guide target IOP but mainly describe current damage and are limited by visual field (VF) variability and OCT floor effects [35,50,57,58,59,60]. AI/electronic health record (EHR) models can estimate surgical risk [63,65,66,67], but clinical use requires external validation, calibration, interpretability, bias auditing, workflow integration, and outcome benefit. PRS may identify high lifetime susceptibility [68,69,70,71], but ancestry portability, cost, privacy, consent, availability, and action thresholds remain unresolved. These approaches are compared in Table 3.

## 4. The Limitations of Medical Therapy

### 4.1. Medication Adherence Challenges

As introduced in Section 1, the traditional medical approach is fundamentally limited by high rates of patient nonadherence and inefficient drug delivery [7,74]. Epidemiological data estimate that up to 80% of patients deviate from their prescribed daily regimens [74]. Barriers to adherence include forgetfulness, poor self-efficacy, difficulty instilling eye drops, and complex multidrug schedules [75]. Poor adherence to topical therapy is a significant predictor of glaucomatous visual field progression [76]. Clinical data indicate that omitting over one-third of prescribed topical doses directly correlates with an increased risk of visual field deterioration [77]. Furthermore, topical drops demonstrate poor bioavailability, with approximately 20% of an individual dose wasted due to inefficient delivery [78].

### 4.2. Ocular Surface Toxicity and Quality of Life (QoL)

Chronic exposure to topical glaucoma medications and their preservatives frequently induces ocular surface disease. The most common preservative, benzalkonium chloride, demonstrates dose-dependent cytotoxicity to conjunctival and corneal epithelial cells [8,35]. Ocular surface disease affects up to 70% of patients on chronic topical therapy [79]. This iatrogenic inflammation manifests as dry eye syndrome and blepharitis, which subsequently diminish patient QoL and exacerbate nonadherence [80]. Most critically, preoperative conjunctival inflammation induced by long-term preservative exposure increases the risk of subconjunctival scarring and early failure of subsequent glaucoma filtration surgeries [81].

### 4.3. Economic and Psychosocial Burden

The lifelong reliance on medical management imposes a substantial socioeconomic burden on patients and healthcare systems. Direct medical costs increase significantly as the disease progresses, with medication expenses constituting the majority of costs in the early stages [82,83]. Glaucoma and its treatments also exert profound psychosocial effects. Patients report decreased vision-related QoL, including reduced mobility, an increased incidence of falls, and a higher risk of motor vehicle collisions [84,85]. The chronic nature of the disease and the daily requirement for drop instillation contribute to elevated rates of anxiety and depression among affected individuals [86]. The cumulative impact of these medical, economic, and psychosocial limitations serves as the primary catalyst for the transition toward earlier surgical interventions [11,87].

## 5. Timing of Surgical Intervention and Proactive Decision-Making

### 5.1. The Timing of Surgery: Proactive Versus Salvage Intervention

The timing of intervention should be proactive but not indiscriminate. Conservative medical therapy or observation remains appropriate for low-risk ocular hypertension, stable mild POAG, low-risk PACS, patients with limited life expectancy, or eyes in which procedural risk exceeds the expected benefit. Earlier laser or surgical intervention becomes reasonable when documented progression, high baseline or peak/diurnal IOP, advanced structural–functional damage, poor adherence, ocular surface toxicity, intolerance, or socioeconomic barriers make medication-dependent control unlikely [35,88]. Evidence from the Collaborative Initial Glaucoma Treatment Study (CIGTS) and primary trabeculectomy for advanced glaucoma study (TAGS) indicates that initial surgery can be an appropriate option for selected patients with advanced open-angle glaucoma (OAG), particularly when very low target IOP is required [88,89]. Conversely, trial findings supporting SLT, MIGS, trabeculectomy, tube shunts, or lens extraction should be applied within their studied populations and should not be generalized to all glaucoma phenotypes. Table 4 summarizes the landmark trials informing interventional decision-making in primary glaucoma.

Collectively, the evidentiary basis for earlier intervention remains uneven. Randomized evidence is strongest for first-line SLT in suitable ocular hypertension or mild-to-moderate OAG, primary trabeculectomy in selected advanced OAG, and clear-lens extraction in EAGLE-eligible PAC or PACG. By contrast, evidence for many MIGS devices and sustained-release platforms is limited by heterogeneous comparators, concurrent cataract surgery, relatively short follow-up, and reliance on IOP or medication reduction rather than visual-field preservation or quality-of-life outcomes. Earlier intervention should therefore not be interpreted as universal procedural escalation; treatment selection must account for glaucoma phenotype, target IOP, procedural risk, durability, cost, patient preference, and preservation of future surgical options.

### 5.2. Establishing Severity-Based Target Intraocular Pressure (IOP)

Once the decision to intervene is made, disease staging helps establish an initial target IOP and follow-up intensity. Target IOP should be individualized and revised over time according to untreated baseline IOP, peak or diurnal fluctuation, CCT and other factors affecting IOP interpretation, severity, rate of progression, age, life expectancy, central-field threat, fellow-eye status, comorbidity, treatment burden, and patient preference [16,35]. The purpose of target IOP is not merely pressure reduction but preservation of visual function with an acceptable safety profile and a rate of progression compatible with maintaining vision-related QoL.

Guidelines commonly recommend an initial IOP reduction of approximately 20% to 30% below untreated baseline, followed by escalation if structural or functional progression continues [35]. The following ranges are practical starting points rather than fixed thresholds:

- Mild (Early) glaucoma: Achieving a target IOP of 18 to 20 mmHg with at least a 20% reduction from baseline may be a reasonable starting point [35]. Patients in this category require less frequent appointments and can be efficiently and safely managed in community-based, multidisciplinary clinics [15,94].
- Moderate glaucoma: A target IOP of 15 to 17 mmHg, typically representing at least a 30% reduction from baseline, is often appropriate, but the target should be lowered if progression continues despite apparently acceptable clinic IOP [35].
- Severe (Advanced) glaucoma: Advanced or rapidly progressing disease may require low-teen or even lower IOP targets, often achievable only with filtering surgery or tube shunt surgery [35,95]. NTG requires particular caution because progression may occur despite statistically normal IOP. Targets should consider nocturnal hypotension, vascular comorbidity, central-field threat, and the risk of hypotony. Patients with severe glaucoma, rapid progression, or only one functional eye must be prioritized and managed in higher-acuity, hospital-based specialty clinics with shortened intervals between follow-up visits [15,35].

## 6. Procedure-Specific Decision-Making and Treatment Algorithms

Table 5 compares the principal interventions and explicitly distinguishes evidence for IOP reduction, medication reduction, visual-field preservation, quality-of-life improvement, and reduced need for later surgery.

### 6.1. Laser Therapy as a Primary Intervention

SLT has emerged as a first-line intervention for suitable patients with POAG and ocular hypertension [90]. The Laser in Glaucoma and Ocular Hypertension (LiGHT) trial demonstrated that initial SLT provided equivalent IOP control and better cost-effectiveness compared with topical medications at 36 months, with 72-month extension data supporting durable disease control and reduced need for subsequent cataract and filtering surgeries [90,91]. These benefits make SLT attractive for patients with poor adherence, ocular surface disease, or a desire to reduce medication burden. Nevertheless, SLT response is variable, may diminish over time, and depends on angle anatomy, trabecular pigmentation, baseline IOP, and the ability to visualize the trabecular meshwork. It should therefore be presented as a first-line option for appropriate ocular hypertension (OHT) or mild-to-moderate OAG, not as a universal replacement for medications or surgery.

### 6.2. The Role of Minimally Invasive Glaucoma Surgery (MIGS)

MIGS encompasses a heterogeneous group of ab interno procedures designed to lower IOP with less tissue trauma and faster recovery than traditional filtering surgery [100]. These procedures target physiological outflow pathways by bypassing the trabecular meshwork into Schlemm’s canal, accessing the suprachoroidal space, or reducing aqueous production [6]. Their main clinical role is in mild-to-moderate OAG, frequently combined with phacoemulsification, where reducing medication burden or achieving mid-teen targets is realistic [16,100]. However, MIGS generally produces less IOP lowering than trabeculectomy or tube shunt surgery and is not appropriate when advanced disease requires very low target IOP [102]. Evidence varies substantially by device, comparator, follow-up duration, and whether cataract surgery is performed concurrently; cost-effectiveness and long-term durability remain important implementation considerations.

### 6.3. Advanced and Refractory Open-Angle Glaucoma (OAG)

Trabeculectomy augmented with antifibrotic agents remains the most established operation for achieving very low target IOP in advanced or rapidly progressing OAG [102]. The Treatment of Advanced Glaucoma Study (TAGS) supports primary trabeculectomy as a viable initial option in newly diagnosed advanced OAG when discussed through shared decision-making [89]. Tube shunts are important alternatives, particularly in eyes with previous ocular surgery, failed trabeculectomy, high scarring risk, or complex conjunctival status [92,93,103]. The Primary Tube Versus Trabeculectomy (PTVT) study and the Tube Versus Trabeculectomy (TVT) study demonstrated that both trabeculectomy and tube shunt surgery can provide substantial long-term IOP reduction, but their relative advantages differ according to prior surgery, medication tolerance, desired target IOP, bleb-related risk, and need for intensive postoperative care [92,93].

### 6.4. Primary Angle-Closure Disease (PACD) Algorithms

**Figure 1:**
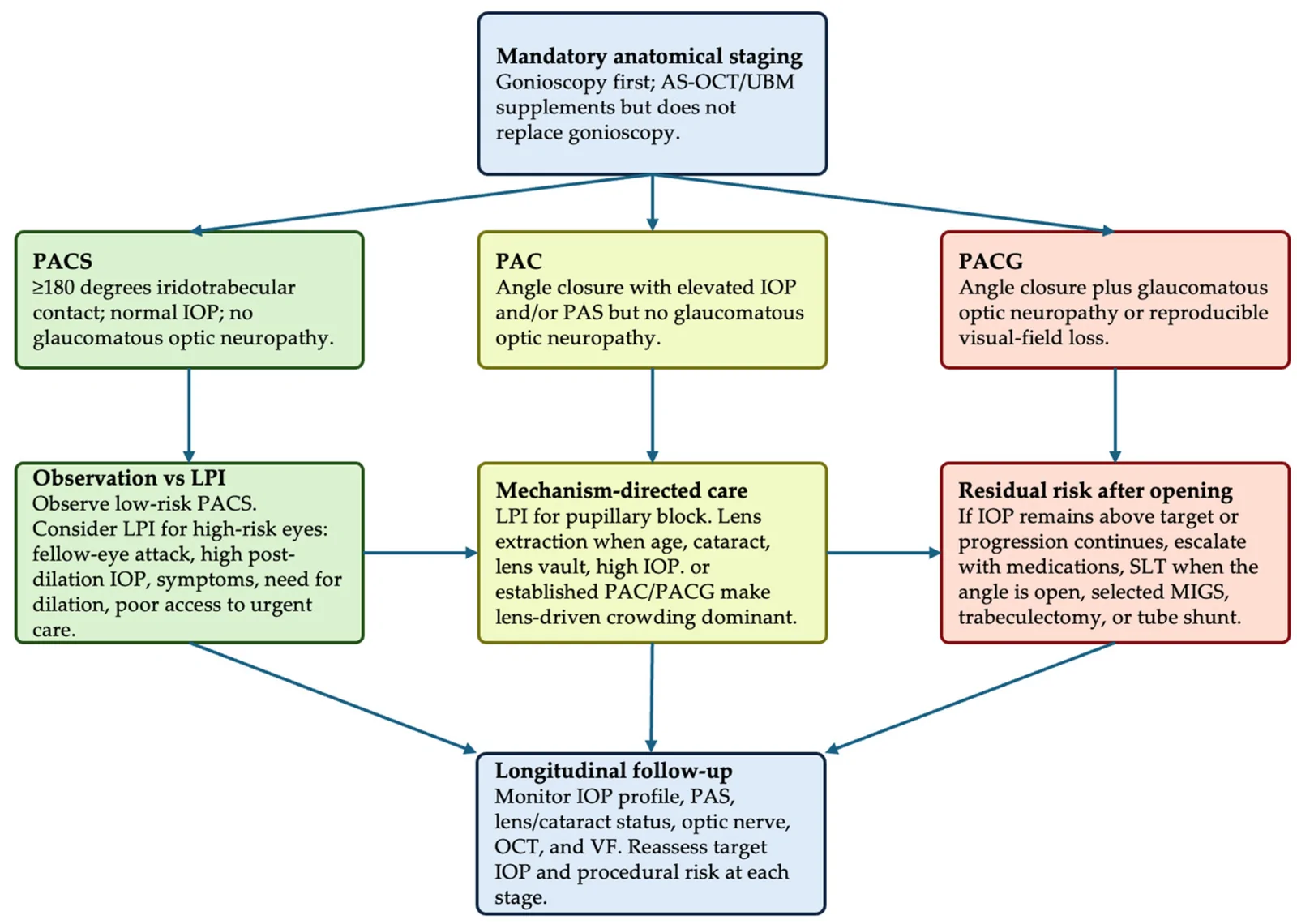
Risk-stratified management algorithm for primary angle-closure disease (PACD). Gonioscopy remains the mandatory first step. Low-risk PACS may be observed, whereas LPI is reserved for high-risk PACS or pupillary-block mechanisms. Lens extraction is favored for selected older PAC or PACG patients when lens-related crowding, cataract, or high IOP supports this approach. Persistent IOP elevation or progression after angle opening requires longitudinal reassessment and escalation according to the residual mechanism and target IOP. AS-OCT, anterior-segment optical coherence tomography; UBM, ultrasound biomicroscopy; PACS, primary angle-closure suspect; IOP, intraocular pressure; PAC, primary angle closure; PACG, primary angle-closure glaucoma; LPI, laser peripheral iridotomy; SLT, selective laser trabeculoplasty; MIGS, minimally invasive glaucoma surgery; PAS, peripheral anterior synechiae; VF, visual field.

PACD management begins with anatomical staging by gonioscopy, followed by mechanism-directed treatment rather than a single procedure for all eyes [35,39,72,73]. Figure 1 summarizes a risk-stratified pathway for PACS, PAC, and PACG.

Progression from PACS to PAC/PACG or acute angle closure is uncommon. Consequently, performing a prophylactic laser peripheral iridotomy (LPI) is no longer universally recommended for all PACS patients [39,73]. LPI remains appropriate for high-risk situations, including fellow-eye acute angle-closure symptoms, high post-dilation IOP, extensive appositional closure, anticipated frequent pharmacologic dilation, or poor access to urgent ophthalmic care [38,39]. For selected patients aged 50 years or older with PAC and IOP ≥ 30 mmHg, or with PACG, the Effectiveness in Angle-Closure Glaucoma of Lens Extraction (EAGLE) trial demonstrated that clear lens extraction produced better health-status and IOP outcomes and was more cost-effective than LPI and topical therapy [72]. This evidence should be applied carefully: cataract status, lens vault, visual needs, surgical risk, and postoperative target IOP all influence the decision, and lens extraction alone may not control IOP when PAS is extensive or advanced optic neuropathy requires lower targets. Residual or progressive disease after angle opening should be treated according to the remaining mechanism, including medications, SLT if the angle is sufficiently open, selected MIGS, trabeculectomy, or tube shunt surgery.

## 7. Patient-Centered Considerations in the Interventional Pathway

### 7.1. Shared Decision-Making and Patient Preferences

Preserving vision-related QoL and advancing general patient welfare stand as the primary objectives of glaucoma care, which must be achieved within an economically and practically viable healthcare system. As discussed in Section 4, the diagnosis of glaucoma, alongside its chronic treatments, profoundly impacts daily activities and induces significant psychosocial anxiety [104,105]. An information gap often exists where patients feel uncertain about clinical systems and treatment outcomes. Bridging this gap requires comprehensive patient education and shared decision-making. Clinicians must provide accurate and accessible written information regarding the disease process, the rationale for intervention, and the pros and cons of different operative procedures. Furthermore, tailoring therapeutic strategies requires physicians to account for the unique socioeconomic realities and personal expectations of each individual. An informed patient is better equipped to participate in management decisions and adhere to postoperative care protocols [16,35].

### 7.2. Mitigating Postoperative Complications

Surgical intervention introduces inherent risks that must be carefully managed to prevent vision loss and maintain QoL. Traditional filtering procedures carry the risk of sight-threatening complications, including hypotony or hypotony maculopathy, choroidal detachment, bleb leak, blebitis, endophthalmitis, cataract acceleration, and long-term failure from subconjunctival scarring [106,107,108]. Tube shunts add device-related concerns such as tube exposure or erosion, corneal endothelial cell loss, motility disturbance, and the need for late revision [92,93]. MIGS generally has a more favorable safety profile, but hyphema, transient IOP spikes, malposition, obstruction, PAS, inflammation, device-specific endothelial cell loss, and other late events must still be discussed [14,100]. Standardized definitions and reporting windows for postoperative complications are essential so that clinicians and patients can compare options honestly across studies and across practice settings [108].

### 7.3. Preserving Future Surgical Options

Glaucoma is a lifelong disease that often requires sequential therapy. Tissue-sparing interventions such as SLT, sustained-release platforms, or selected MIGS may reduce medication burden and preserve conjunctiva for later filtering surgery, but this principle should not justify delaying definitive trabeculectomy or tube shunt surgery when advanced disease is progressing toward fixation [11,81,101,109]. Prior angle-based or intraocular procedures may nevertheless induce PAS, alter anatomy, or affect subsequent surgery. Phacoemulsification combined with an appropriate glaucoma procedure may delay more invasive surgery in selected eyes, but cataract-related improvement in visual function can confound patient-reported outcomes and should be distinguished from true glaucoma stabilization [101]. The key is longitudinal escalation. After inadequate SLT or MIGS, options include repeat SLT where appropriate, medication, or filtration/tube surgery according to target IOP. Failed filtering or tube procedures require mechanism-specific reassessment and may necessitate revision or alternative incisional or cyclodestructive treatment [35,92,93,101]. Each procedure should be chosen for the current target IOP while preserving reasonable future options.

### 7.4. Implementation Across Resource Settings and Health Equity

Risk-stratified care must be feasible in the setting where it is delivered. In high-resource systems, OCT, perimetry, SLT, MIGS, sustained-release platforms, and subspecialty surgery may allow highly individualized escalation. In lower-resource settings, gonioscopy, optic nerve examination, IOP measurement, visual acuity, pragmatic visual-field testing, low-cost risk tools, adherence support, and reliable referral pathways may be more important than device-intensive precision. Disparities in glaucoma surgery can occur at patient selection, procedure type, timing, complication management, follow-up, and long-term outcomes [110]. Therefore, interventional algorithms should explicitly consider insurance status, travel distance, disability, language, health literacy, medication affordability, and the ability to attend intensive postoperative visits.

## 8. Future Directions and Unmet Needs

### 8.1. Precision Medicine and Biomarkers

As introduced in Section 3.4, PRS may eventually personalize surveillance and treatment timing by identifying patients with high lifetime susceptibility, earlier onset, or faster progression [68,69,70,71]. At present, however, PRS should be considered investigational adjuncts rather than stand-alone indications for surgery. Barriers include limited transferability across ancestries, uncertainty about how much PRS changes management beyond clinical risk factors, cost, reimbursement, genetic counseling capacity, data privacy, consent, and the risk of widening inequities if testing is available only to selected populations. Beyond genomics, molecular biomarkers of conjunctival fibrosis and wound healing remain a critical unmet need because they could guide antifibrotic selection, follow-up intensity, or procedure choice [111,112].

### 8.2. Sustained-Release Drug Delivery Platforms

To overcome the limitations of topical therapies, as discussed in Section 4, novel sustained-release drug delivery platforms are rapidly advancing [74]. Intracameral implants eluting bimatoprost or travoprost demonstrate IOP reductions comparable to topical therapies over extended periods [96,97,98,99]. Other emerging platforms under clinical investigation include extracameral rings, punctal plugs, and drug-eluting contact lenses [113]. Nanotechnology-based platforms employing polymeric hydrogels and liposomes offer potential avenues to overcome ocular structural barriers and increase drug bioavailability [114]. However, long-term clinical trials must rigorously evaluate the cytotoxicity and pharmacokinetic profiles of these devices before widespread clinical adoption occurs.

### 8.3. Artificial Intelligence (AI) and Standardized Outcomes

AI and deep learning models can assist with diagnosis, progression detection, and prediction of future surgery [62,67,115]. However, robust clinical implementation requires more than high internal accuracy. Models should be externally validated across devices, ethnic groups, disease stages, imaging protocols, and health systems; calibrated to clinically meaningful decision thresholds; governed for privacy, regulatory oversight, and clinical accountability; monitored for drift; and evaluated prospectively to show that their use improves outcomes or resource allocation. Missing EHR data, referral bias, treatment-selection bias, inconsistent endpoints, and opaque model reasoning can all reduce real-world reliability. For these reasons, AI should currently support, rather than replace, clinician judgment and shared decision-making. Harmonized core outcome sets are also needed so future trials define surgical success, failure, complications, visual preservation, and patient-reported outcomes consistently [116]. Longer-term, adequately powered head-to-head trials are needed to compare procedure-specific effects on VF preservation, medication burden, QoL, reoperation, and cost-effectiveness across representative patient populations.

### 8.4. Current Controversies and Research Gaps

The optimal timing of glaucoma surgery remains controversial. Earlier intervention may improve IOP control and reduce medication burden but may expose patients with stable disease to unnecessary procedural risk. Evidence for MIGS is limited by device heterogeneity, concurrent cataract surgery, short follow-up, and reliance on IOP rather than visual-field or patient-centered outcomes [100,101,102]. The comparative durability, reoperation rates, QoL effects, and cost-effectiveness of available procedures therefore remain uncertain. AI and PRS also require external validation, calibration, interpretability, and equitable implementation before informing surgical decisions [62,67,68,69,70,71,115]. Disparities in access and postoperative care may further limit generalizability [110]. Future studies should use standardized outcome definitions and adequately powered, long-term head-to-head designs evaluating visual field preservation, medication burden, quality of life, reoperation, and cost-effectiveness [108,116].

## 9. Conclusions

Primary glaucoma requires lifelong risk-adapted management to preserve visual function while minimizing treatment burden. Because IOP remains the only proven modifiable risk factor, target pressure should be individualized and repeatedly revised according to structural–functional stage, rate of progression, ocular phenotype, life expectancy, adherence, patient preference, and resource setting.

For suitable OHT and mild-to-moderate OAG, SLT, medications, sustained-release therapy, and selected MIGS can reduce IOP and medication burden. For advanced or rapidly progressing OAG, trabeculectomy or tube shunt surgery should be considered when lower target pressures are required. For PACD, gonioscopy-based anatomical staging is essential; low-risk PACS can often be observed, while LPI, lens extraction, or glaucoma surgery should be selected according to mechanism and residual risk.

AI, PRS, and biomarkers may eventually refine risk prediction and procedural selection, but they require external validation, equitable implementation, and clinically actionable thresholds before routine use. A balanced interventional framework should therefore combine evidence from landmark trials with patient-centered judgment, postoperative safety, and practical feasibility.

## Figures and Tables

**Table 1:** Risk factors for the onset and progression of primary open-angle glaucoma.

Risk Category	Specific Risk Factor	Pathophysiological Impact and Clinical Significance	Reference
Demographic Factors	Older age	Increased disease prevalence and faster visual field deterioration	[2]
	African, Hispanic, or Latino ancestry	Earlier disease onset and higher rates of severe visual impairment	[21,22,23]
	Family history of glaucoma	First-degree relatives of affected individuals with elevated risk of disease development	[24]
	Male sex	Higher risk of glaucoma onset	[23]
Ocular Factors	Elevated IOP ^1^	The primary modifiable risk factor driving RGC ^2^ apoptosis and structural progression	[25]
	Thinner CCT ^3^	Predicts conversion from ocular hypertension to glaucoma and affects tonometry interpretation	[26]
	Lower corneal hysteresis	Indicates reduced viscoelastic damping and predicts progression independently of IOP	[27,28]
	Myopia	Confers structural susceptibility and increases the risk of POAG ^4^ onset	[29,30]
	Disc hemorrhage	Active focal structural damage predicting future localized visual field loss	[31]
	Larger CDR ^5^	Reflects baseline structural RGC loss and predicts further structural and functional decline	[20,32]
	High PSD ^6^	Baseline visual-field irregularity correlates with subsequent functional deterioration	[33,34]
	Pseudoexfoliation syndrome	Fibrillar protein deposition increases outflow resistance and is linked to rapid disease progression	[35]
Systemic Factors	Low OPP ^7^	Compromised optic nerve head perfusion may increase susceptibility to structural damage and functional loss	[36]
	Low systemic blood pressure	Systemic hypotension impairs adequate ocular perfusion and increases progression risk	[36]
	T2DM ^8^	Microvascular alterations may increase optic nerve vulnerability and disease prevalence	[37]

^1^ IOP, intraocular pressure; ^2^ RGC, retinal ganglion cell; ^3^ CCT, central corneal thickness; ^4^ POAG, primary open-angle glaucoma; ^5^ CDR, cup-to-disc ratio; ^6^ PSD, pattern standard deviation; ^7^ OPP, ocular perfusion pressure; ^8^ T2DM, type 2 diabetes mellitus.

**Table 2:** Risk factors for the primary angle-closure disease spectrum.

Risk Category	Clinical and Pathophysiological Details	Reference
Demographic Risk Factors	Advancing age	[2,35,40,41]
	Female sex	
	Asian or Inuit ancestry (East Asian and Chinese populations)	
Ocular Biometric and Structural Factors	Shallow anterior chamber depth	[35,42,43]
	Shorter axial length (hypermetropia)	
	Thicker peripheral iris with a more anterior insertion	
	Prominent, anteriorly vaulted crystalline lens	
	The continuous, age-related increase in lens volume and thickness	
Dynamic and Physiological Mechanisms	Choroidal expansion	[35,44]
	Abnormal zonular biomechanics and laxity (e.g., pseudoexfoliation syndrome)	
Genetic Susceptibility	Family history of angle-closure	[45,46]
	Specific loci, including PLEKHA7, COL11A1 (Stickler syndrome), and EPDR1	
Environmental and Iatrogenic Triggers	Systemic medication-induced mydriasis (e.g., sympathomimetics, anticholinergic agents, and specific antidepressants like selective serotonin reuptake inhibitors)	[47,48,49]

**Table 3:** Comparative summary of glaucoma risk stratification models and clinical decision tools.

Model or Tool	Core Inputs	Strengths	Limitations and Clinical Applicability	Reference
EGPS-OHTS ^1^ calculator	Age, IOP ^2^, CCT ^3^, vertical CDR ^4^, PSD ^5^	Transparent and validated for ocular hypertension; useful for counseling and treatment thresholds	Predicts conversion, not established glaucoma progression; limited for PACD ^6^, NTG ^7^, and populations unlike derivation cohorts.	[33]
Structural–functional staging	SAP MD/VFI ^8^, RNFL/GCC OCT ^9^, DDLS ^10^, optic nerve findings	Clinically familiar; links severity to target IOP and follow-up intensity	Reflects current damage more than future risk; affected by VF ^11^ variability, OCT artifacts, and floor effects.	[35,50,57,58,59,60]
GLAUC-STRAT and red-flag triage	Severity, progression rate, IOP, one-eye status, access or socioeconomic risk	Useful for clinic prioritization and resource allocation	Broad categories require local validation and adaptation to workforce and access constraints.	[61]
AI/EHR ^12^ prediction models	Imaging, VF, IOP, medications, procedures, demographics, EHR variables	Can capture nonlinear multimodal patterns and estimate surgery risk	Needs external/prospective validation, calibration, explainability, privacy governance, and bias auditing.	[63,65,66,67]
PRS ^13^ and genetic profiling	Common risk variants, selected rare variants, ancestry context	May identify high lifetime susceptibility and early-onset risk	Action thresholds, cost-effectiveness, ancestry portability, consent, and equity remain unresolved.	[68,69,70,71]
PACD anatomic risk assessment	Gonioscopy, PAS ^14^, AS-OCT/UBM ^15^, lens vault, ACD ^16^, post-dilation IOP	Directly identifies mechanism and guides LPI ^17^ vs. lens-based approaches.	Progression from PACS ^18^ is often low; imaging cannot replace gonioscopy; decisions depend on age, lens/cataract, IOP, and optic nerve status.	[35,39,72,73]

^1^ EGPS-OHTS, European Glaucoma Prevention Study and Ocular Hypertension Treatment Study; ^2^ IOP, intraocular pressure; ^3^ CCT, central corneal thickness; ^4^ CDR, cup–disc ratio; ^5^ PSD, pattern standard deviation; ^6^ PACD, primary angle-closure disease; ^7^ NTG, normal-tension glaucoma; ^8^ SAP MD/VFI, standard automated perimetry mean deviation/visual field index; ^9^ RNFL/GCC OCT, retinal nerve fiber layer/ganglion cell complex optical coherence tomography; ^10^ DDLS, disc damage likelihood scale; ^11^ VF, visual field; ^12^ AI/EHR, artificial intelligence/electronic health records; ^13^ PRS, polygenic risk score; ^14^ PAS, peripheral anterior synechiae; ^15^ AS-OCT/UBM, anterior-segment optical coherence tomography/ultrasound biomicroscopy; ^16^ ACD, anterior chamber depth; ^17^ LPI, laser peripheral iridotomy; ^18^ PACS, primary angle-closure suspect.

**Table 4:** Landmark trials informing interventional decision-making in primary glaucoma.

Trial or Study	Population & Comparison	Key Outcome	Clinical Implication	Reference
OHTS/EGPS ^1^	Ocular hypertension; observation or topical therapy; risk-model development	Age, IOP ^2^, CCT ^3^, vertical CDR ^4^, and PSD ^5^ predicted conversion to POAG ^6^	Supports individualized treatment thresholds in ocular hypertension rather than treating all patients uniformly.	[20,32,33,34]
LiGHT ^7^	Newly treated OHT/OAG ^8^; first-line SLT ^9^ vs. topical drops	SLT achieved durable IOP control, reduced drop burden, and favorable cost-effectiveness	Supports first-line SLT in suitable OHT and mild-to-moderate OAG.	[90,91]
CIGTS ^10^	Newly diagnosed OAG; initial surgery vs. initial medical therapy	Long-term visual-field outcomes were broadly comparable, with lower IOP after surgery	Supports surgery as a legitimate early option in selected advanced or high-risk OAG.	[88]
TAGS ^11^	Newly diagnosed advanced OAG; primary trabeculectomy vs. medical therapy	Trabeculectomy achieved lower IOP with acceptable safety and patient-reported outcomes	Primary trabeculectomy should be discussed for advanced OAG when low targets are needed.	[89]
PTVT/TVT ^12^	Eyes requiring incisional surgery; tube shunt vs. trabeculectomy with MMC ^13^	Both approaches lowered IOP; relative advantages varied by prior surgery and medication need	Procedure choice should consider prior surgery, target IOP, medication burden, and bleb-related risk.	[92,93]
ZAP/ANA-LIS ^14^	Asymptomatic PACS ^15^; LPI ^16^ vs. observation	Absolute progression risk was low in many PACS eyes	Supports selective rather than universal prophylactic LPI in low-risk PACS.	[38,39,73]
EAGLE ^17^	Selected PAC/PACG ^18^ patients aged ≥50 years; clear lens extraction vs. LPI/medical care	Lens extraction improved IOP-related outcomes and cost-effectiveness in the studied population	Supports early lens extraction for selected older PAC/PACG patients, not blanket treatment of all PACD ^19^.	[72]

^1^ OHTS/EGPS, Ocular Hypertension Treatment Study/European Glaucoma Prevention Study; ^2^ IOP, intraocular pressure; ^3^ CCT, central corneal thickness; ^4^ CDR, cup–disc ratio; ^5^ PSD, pattern standard deviation; ^6^ POAG, primary open-angle glaucoma; ^7^ LiGHT, Laser in Glaucoma and Ocular Hypertension; ^8^ OHT/OAG, ocular hypertension/open-angle glaucoma; ^9^ SLT, selective laser trabeculoplasty; ^10^ CIGTS, Collaborative Initial Glaucoma Treatment Study; ^11^ TAGS, Treatment of Advanced Glaucoma Study; ^12^ PTVT/TVT, Primary Tube Versus Trabeculectomy/Tube Versus Trabeculectomy; ^13^ MMC, mitomycin C; ^14^ ZAP/ANA-LIS, Zhongshan Angle-Closure Prevention/Asymptomatic Narrow Angle Laser Iridotomy Study; ^15^ PACS, primary angle-closure suspect; ^16^ LPI, laser peripheral iridotomy; ^17^ EAGLE, Effectiveness in Angle-Closure Glaucoma of Lens Extraction; ^18^ PAC/PACG, primary angle-closure/primary angle-closure glaucoma; ^19^ PACD, primary angle-closure disease.

**Table 5:** Practical comparison of principal interventions and evidence-supported outcomes in primary glaucoma.

Intervention	Best-Fit Clinical Role	Evidence-Supported Outcomes	Limitations and Cautions	Reference
Medication	Low-risk or stable disease; adjunctive therapy after procedures	**IOP** ^1^ **reduction:** Established. **Medication reduction:** Not an intended outcome. **VF** ^2^ **preservation:** Supported at the treatment-strategy level when pressure control is sustained, but not inferred from a single IOP response. **QoL** ^3^ **improvement:** Not consistently demonstrated; treatment burden and adverse effects may reduce QoL. **Reduced later surgery:** Not established; medical control may defer, but does not necessarily prevent, surgery.	Adherence, ocular surface toxicity, cost, technique errors, and limited effect of clinic IOP on peak/diurnal IOP.	[20,77,79,80,88]
SLT ^4^	OHT ^5^ or mild-to-moderate OAG ^6^ with visible trabecular meshwork	**IOP reduction:** Established. **Medication reduction:** Established. **VF preservation:** Disease control is comparable to initial drops; superiority for VF preservation is not established. **QoL improvement:** No clear superiority over drops. **Reduced later surgery:** Supported for filtering surgery in the 6-year LiGHT ^7^ follow-up.	Variable response, waning effect, less suitable when very low target IOP is required.	[90,91]
Sustained-release therapy	Patients limited by adherence or ocular surface toxicity	**IOP reduction:** Established over short-to-intermediate follow-up. **Medication reduction:** Supported while the implant remains effective. **VF preservation:** Long-term benefit is not established. **QoL improvement:** Not established. **Reduced later surgery:** Not established.	Long-term safety, retreatment strategy, cost, and real-world durability require continued evaluation.	[96,97,98,99]
MIGS ^8^	Mild-to-moderate OAG, commonly with cataract surgery	**IOP reduction:** Supported, generally less than with filtering surgery. **Medication reduction:** Supported, particularly when MIGS is combined with cataract surgery. **VF preservation:** Not established by robust long-term comparative evidence. **QoL improvement:** Not established independently of concurrent cataract surgery. **Reduced later surgery:** Not established.	Less IOP lowering than trabeculectomy or tube shunt; device-specific evidence and cost vary.	[100,101,102]
Lens extraction in PACD ^9^	Selected PAC/PACG ^10^ with lens-driven crowding, cataract, or older age	**IOP reduction:** Supported in selected patients meeting EAGLE ^11^ eligibility criteria. **Medication reduction:** Supported in the studied population. **VF preservation:** Long-term preservation is not established. **QoL improvement:** Supported in the EAGLE population. **Reduced later surgery:** Not established as a durable glaucoma-specific benefit.	Not sufficient for all eyes; residual PAS ^12^, advanced optic neuropathy, or high target demands may require additional glaucoma procedures.	[72]
Trabeculectomy	Advanced or rapidly progressing disease requiring low target IOP	**IOP reduction:** Established, including achievement of low target IOP. **Medication reduction:** Established. **VF preservation:** CIGTS ^13^ found broadly comparable long-term VF outcomes with initial surgery and medication; superiority is not established. **QoL improvement:** No superiority at 2 years in TAGS ^14^. **Reduced later surgery:** Not consistently demonstrated; bleb revision or cataract surgery may be required.	Bleb-related infection, hypotony, intensive postoperative care, scarring, and cataract progression.	[88,89]
Tube shunt	Prior failed filtering surgery, high scarring risk, or selected primary incisional cases	**IOP reduction:** Established. **Medication reduction:** Supported, although medication need may exceed trabeculectomy in some primary cases. **VF preservation:** No consistent advantage over trabeculectomy. **QoL improvement:** Not established as a comparative benefit. **Reduced later surgery:** Failure and reoperation outcomes vary by population; no universal reduction is established.	May require more medications than trabeculectomy in some primary cases; tube position, corneal risk, and diplopia require surveillance.	[92,93]

^1^ IOP, intraocular pressure; ^2^ VF, visual field; ^3^ QoL, quality of life; ^4^ SLT, selective laser trabeculoplasty; ^5^ OHT, ocular hypertension; ^6^ OAG, open-angle glaucoma; ^7^ LiGHT, Laser in Glaucoma and Ocular Hypertension; ^8^ MIGS, minimally invasive glaucoma surgery; ^9^ PACD, primary angle-closure disease; ^10^ PAC/PACG, primary angle closure/primary angle-closure glaucoma; ^11^ EAGLE, Effectiveness in Angle-Closure Glaucoma of Lens Extraction; ^12^ PAS, peripheral anterior synechiae; ^13^ CIGTS, Collaborative Initial Glaucoma Treatment Study; ^14^ TAGS, Treatment of Advanced Glaucoma Study.

## Data Availability

The original contributions presented in this study are included in the article. Further inquiries can be directed to the corresponding authors.

## Abbreviations

The following abbreviations are used in this manuscript:

RGC	retinal ganglion cell
POAG	primary open-angle glaucoma
PACG	primary angle-closure glaucoma
IOP	intraocular pressure
QoL	quality of life
SLT	selective laser trabeculoplasty
MIGS	minimally invasive glaucoma surgery
CCT	central corneal thickness
PACD	primary angle-closure disease
PAS	peripheral anterior synechiae
AS-OCT	anterior-segment optical coherence tomography
ZAP	Zhongshan Angle-Closure Prevention
ANA-LIS	Asymptomatic Narrow Angle Laser Iridotomy Study
PACS	primary angle-closure suspect
PAC	primary angle closure
MD	mean deviation
VFI	visual field index
mGSS	modified Glaucoma Staging System
RNFL	retinal nerve fiber layer
GCC	ganglion cell complex
GGSS	Global Glaucoma Staging System
SAP	standard automated perimetry
AI	artificial intelligence
HPA	Hodapp–Parrish–Anderson
DDLS	Disc Damage Likelihood Scale
ITC	iridotrabecular contact
GLAUC-STRAT	Glaucoma Stratification
CDR	cup-to-disc ratio
EGPS-OHTS	European Glaucoma Prevention Study and Ocular Hypertension Treatment Study
NTG	normal-tension glaucoma
VF	visual field
EHR	electronic health records
PRS	polygenic risk scores
CIGTS	Collaborative Initial Glaucoma Treatment Study
TAGS	Treatment of Advanced Glaucoma Study
OAG	open-angle glaucoma
LiGHT	Laser in Glaucoma and Ocular Hypertension
OHT	ocular hypertension
PTVT	Primary Tube Versus Trabeculectomy
TVT	Tube Versus Trabeculectomy
LPI	laser peripheral iridotomy
EAGLE	Effectiveness in Angle-Closure Glaucoma of Lens Extraction
